# Activation of Stress Kinases in the Brain of Mucopolysaccharidosis IIIB mice

**DOI:** 10.1002/jnr.22674

**Published:** 2011-05-26

**Authors:** Francesca Cecere, Carmela Di Domenico, Daniele Di Napoli, Francesca Boscia, Paola Di Natale

**Affiliations:** 1 Department of Biochemistry and Medical Biotechnologies, University of Naples Federico IINaples, Italy; 2 Center for Animal Experimentation, Cardarelli HospitalNaples, Italy; 3 Department of Neuroscience, University of Naples Federico IINaples, Italy

**Keywords:** mucopolysaccharidosis, neurodegeneration, knockout mouse

## Abstract

The accumulation of heparan sulfate (HS) in lysosomes is the primary consequence of the enzyme defect (α-N-acetylglucosaminidase) in mucopolysaccharidosis type IIIB. This accumulation triggers a cascade of pathological events that progressively leads to CNS pathology. Here we examined the activation of the three major stress kinases in the neuronal tissue of a murine model of the disease. ERK1/2 was significantly higher in the cortex of 1–2-month-old affected animals compared with wild-type (Wt) littermates. Similarly, ERK1/2 was stimulated in neurons cultured from MPS IIIB mice. SAPK/JNK was also found to be activated in the cortex of 1–2-month-old affected animals compared with Wt subjects, and the same was found for cultured neurons. In contrast, the active form of p38MAPK was lower in the cortex of 1-month-old MPS IIIB mice compared with Wt animals, but no significant difference was found between the two p38MAPK analyzed in normal and affected neurons cultured in vitro. These data indicate the possible involvement of MAPK dysregulation in the early stage of MPS IIIB brain disease. © 2011 Wiley-Liss, Inc.

Mucopolysaccharidosis IIIB (MPS IIIB) is a lysosomal storage disorder (LSD) associated with severe and progressive neurodegeneration, which is the most prominent clinical symptom (Neufeld and Muenzer,^2001^) and the most difficult challenge for therapy (Cressant et al.,^2004^; Zheng et al.,^2004^; Di Natale et al.,^2005^; Di Domenico et al.,^2009^). As for the majority of LSDs, in MPS IIIB the deficiency of a single, specific lysosomal hydrolase (α-N-acetylglucosaminidase, NAGLU; E.C. 3.2.1.50) causes the accumulation in the patient's cells of its undegraded substrate, heparan sulfate (HS). The buildup of this nondegraded substrate is believed to be primarily responsible for the disease pathogenesis. Many factors have been shown to contribute: an inflammatory response with microglial activation followed by the secretion of toxic cytokines (Li et al.,^2002^; Ohmi et al.,^2003^; Villani et al.,^2007^), an alteration in neuroplasticity (Li et al.,^2002^) and neurotrophins (Villani et al.,^2007^), accumulation of immunoreactive subunit c of mitochondrial ATP synthase (Ryazantsev et al.,^2007^), degradation of synaptophysin by the proteasome (Vitry et al.,^2009^), and involvement of T-cell-mediated cytotoxicity (DiRosario et al.,^2009^; Villani et al.,^2009^). However, despite all the efforts to clarify the MPS IIIB disease pathogenesis, the sequence of events from the enzyme deficiency to the cellular dysfunction is still obscure.

The mammalian family of mitogen-activated protein kinases (MAPKs) includes the so-called stress kinases: extracellular signal-regulated kinase (ERK), c-Jun NH2 terminal kinase [JNK; also known as stress-activated protein kinase (SAPK)], and p38 (Seger and Krebs,^1995^; Kim and Choi,^2010^). MAPKs are activated by phosphorylation on specific Thr and Tyr sites by upstream kinases. Extracellular regulated kinase 1/2 (ERK1/2) typically regulates growth and cell proliferation and differentiation, whereas stress-activated protein kinase/Jun kinase (JNK) and p38MAPK are activated in response to osmotic shock, UV irradiation, inflammatory cytokines, and oxidative stress (Haddad and Adams,^2004^). Moreover, JNK has been associated with neurofibrillary pathology (Willaime-Morawek et al.,^2003^), and its up-regulation has been shown to overlap with phosphorylated tau (Zhu et al.,^2001^). Once activated, these serine-threonine kinases mediate a variety of cellular responses, including cell proliferation, differentiation, survival, death, and transformation (McCubrey et al.,^2006^; Dhillon et al.,^2007^). However, although it is well established that ERK activation has beneficial effects on the cells, recent findings with neural cells show that ERK may exert an opposite effect, in that its activation can either promote neuron survival or cause cell death. For example, in a number of neural cell systems, it has been shown that chronic activation of ERK is able to impair the physiological response to neurotrophic factors and/or to drive cells to death (Colucci-D'Amato et al.,^2003^). Moreover, ERK activation was suggested to play a role in a caspase-independent mechanism of cell death involving an alteration of the plasma membrane (Subramaniam et al.,^2004^).{FN0}

We found oxidative stress to be one of the main factors implicated in MPS IIIB disease pathogenesis (Villani et al.,^2007^, ^2009^). One of the consequences of increased reactive oxygen species (ROS) levels in the brain of affected mice might be the activation of the stress kinases. To test this hypothesis, the present study analyzed the expression of phospho-ERK1/2, -SAPK/JNK, and -p38MAPK in the cortex and embryonic neurons cultured from MPS IIIB mice.

## MATERIALS AND METHODS

### Chemicals

Hanks' balanced salt solution and MEM were purchased from Gibco (Grand Island, NY). Fetal bovine serum, L-glutamine, penicillin G, and streptomycin were obtained from Cambrex. Cell Signaling Technology (Beverly, MA) supplied the first antibodies raised against phospho-ERK1/2 (product No. 4370), phospho-SAPK/JNK (product No. 4668), and phospho-p38MAPK (product No. 9211) and the goat anti-rabbit IgG horseradish peroxidase (HRP)-conjugated secondary antibody (product No. 7074). Poly-D-lysine, cytosine arabinoside, and trypsin were purchased from Sigma (Milan, Italy), as were the mouse anti-γ-tubulin monoclonal primary antibody (product No. T6557) and the goat anti-mouse IgG HRP-conjugated secondary antibody.

### Animals

The mice were genotyped by PCR performed on DNA samples extracted from tail biopsy at 1 month after birth, as previously described (Di Natale et al.,^2005^). For the study on adult mouse cortices, homozygous mutant mice and wild-type (Wt) control mice of 1, 2, 3, and 6 months of age were used. At the time of killing, the mice were sacrificed by cervical dislocation after profound anesthesia. The cerebrum was rapidly removed, and the cortex isolated by using a stereomicroscope (Zeiss) on an ice-cold tissue culture plate. The cortices were then transferred to microcentrifuge tubes and immediately stored at –80°C until use. For the preparation of primary neuronal cultures, timed pregnancies of MPS IIIB and Wt mice were set up, and embryos were obtained by cesarean section of pregnant mice 16 days postcoitum. All experimental protocols were approved by the Italian Ministry of Public Health.

### Western Blotting

Brain cortices were homogenized on ice in lysis buffer (20 mM Tris-HCl, pH 7.5, 150 mM NaCl, 1 mM Na_2_EDTA, 1 mM EGTA, 1% Triton, 2.5 mM sodium pyrophosphate, 1 mM β-glycerophosphate, 1 mM Na_3_VO_4_) supplemented with protease inhibitors (1 μg/ml leupeptin, 1 μg/ml aprotinin, 1 mM PMSF) and incubated for 30 min on ice. After centrifugation at 14,000 rpm at 4°C for 30 min, the supernatants were used for protein determination by the Bradford method (Bradford,^1976^) with BSA as a standard. The same protocol was used for the preparation of total protein extract from neuronal cells. Western blotting analysis was carried out on equal amounts of protein extracts. Briefly, 30 μg of total protein extract was boiled for 5 min in SDS/reducing loading buffer, run on a 10% SDS-PAGE, and then transferred onto nitrocellulose membranes (Millipore, Bedford, MA). The membranes were blocked for 1 hr at room temperature with 5% BSA (or 5% skim-milk powder for anti-γ-tubulin) in T-TBS (30 mM Tris-HCl, 125 mM NaCl, 0.1% Tween 20). The blots were then incubated overnight at 4°C with the specific primary antibody, appropriately diluted in T-TBS containing 5% BSA for the phospho-MAPK antibodies or 2% milk for the tubulin antibody. The primary antibodies used were rabbit antiphospho-ERK1/2 (1:1,000), rabbit antiphospho-SAPK/JNK (1:500), rabbit antiphospho-p38MAPK (1:500), and mouse anti-γ-tubulin (1:10,000). After washing in T-TBS (30 mM Tris-HCl, 125 mM NaCl, 0.05% Tween 20), the membranes were incubated for 1 hr at room temperature with goat anti-rabbit or anti-mouse HRP-conjugated secondary antibody diluted in T-TBS containing 2% milk (1:3,000). The proteins were visualized by an enhanced chemiluminescence reaction using ECL detection reagent (Amersham, Arlington Heights, IL), followed by exposure to Fuji film. The same blots were stripped and reprobed with γ-tubulin monoclonal antibody to confirm equal loading of proteins in all lanes. Films were scanned using an HP scanner and subjected to densitometric analysis in ImageJ software.

### Primary Cortical Neuronal Cultures

Neuronal cultures were prepared from embryonic day 16 (E16) mouse cortices as described by Scorziello and colleagues (2001), with some modifications. Brains of mouse embryos, obtained from time-pregnant MPS IIIB and Wt mice, were collected and placed in ice-cold Hanks' balanced salt solution supplemented with 5 mg/ml glucose, 7 mg/ml saccharose, and 0.3 mg/ml sodium bicarbonate. Cortices were then isolated under a stereomicroscope by removing the attached meninges and incubated with 0.25% trypsin for 20 min at 37°C. After centrifugation at 600*g* for 5 min, the cells were dissociated by careful titration through a constricted Pasteur pipette. Dissociated neurons were resuspended in minimal essential medium (MEM) containing glucose, 5% heat-inactivated fetal bovine serum, 5% heat-inactivated horse serum, 2 mM L-glutamine, and 100 U/ml and 100 μg/ml of a mixture of penicilin-streptomycin and seeded at a density of 2 × 10^6^ cells on 35-mm Petri dishes precoated overnight with 0.1 mg/ml poly-D-lysine. After 24 hr, the medium was replaced with freshly prepared medium of similar composition, and neuronal cells were maintained in a humidified 5% CO_2_/95% air atmosphere at 37°C until use. Cytosine-D-arabino-furanoside (10 μM) was added at 5 days in vitro (DIV) to prevent glial proliferation, and experiments were performed on cultures at 8 DIV. The purity of the cultures was verified to be 95% using the neuron-specific marker microtubule-associated protein-2 (MAP2) and the astrocyte marker glial fibrillary acidic protein (GFAP). The data from each experiment were obtained from at least three individual culture preparations (i.e., one embryo was used for one culture preparation), and each experiment was repeated at least three times.

### Measure of HS Levels in Cultured Neurons

The level of HS in embryonic neurons cultured from MPS IIIB and Wt animals was measured according to .sson (1993), with some modifications. Briefly, the total extract from neuron pellets (300 μg) was resuspended in 0.9% NaCl/0.2% Triton X-100, rotated at 4°C overnight, and centrifuged to remove debris. GAGs were precipitated with Alcian blue, and absorbance was measured at 600 nm. HS from porcine intestinal mucosa was used as a standard.

### Statistical Analysis

All results are presented as histograms, and data are expressed as means and SD of five independent experiments. Student's *t*-test was used to assess whether there were significant differences between MPS IIIB and control mice.

## RESULTS

Our work was aimed at the dissection of the intracellular pathways that might be switched on/off in the cortex and in cultured neurons of MPS IIIB mice. Three major protein kinase pathways have been shown to be responsive to insults such as stress, ERK1/2, JNK, and p38MAPK. Therefore, we examined by Western blot the level of the three MAPKs by using antibodies specific for each phosphorylated form of these enzymes.

### ERK and JNK Activation Is Increased in the Cortex of MPS IIIB Mice

**Fig. 1:**
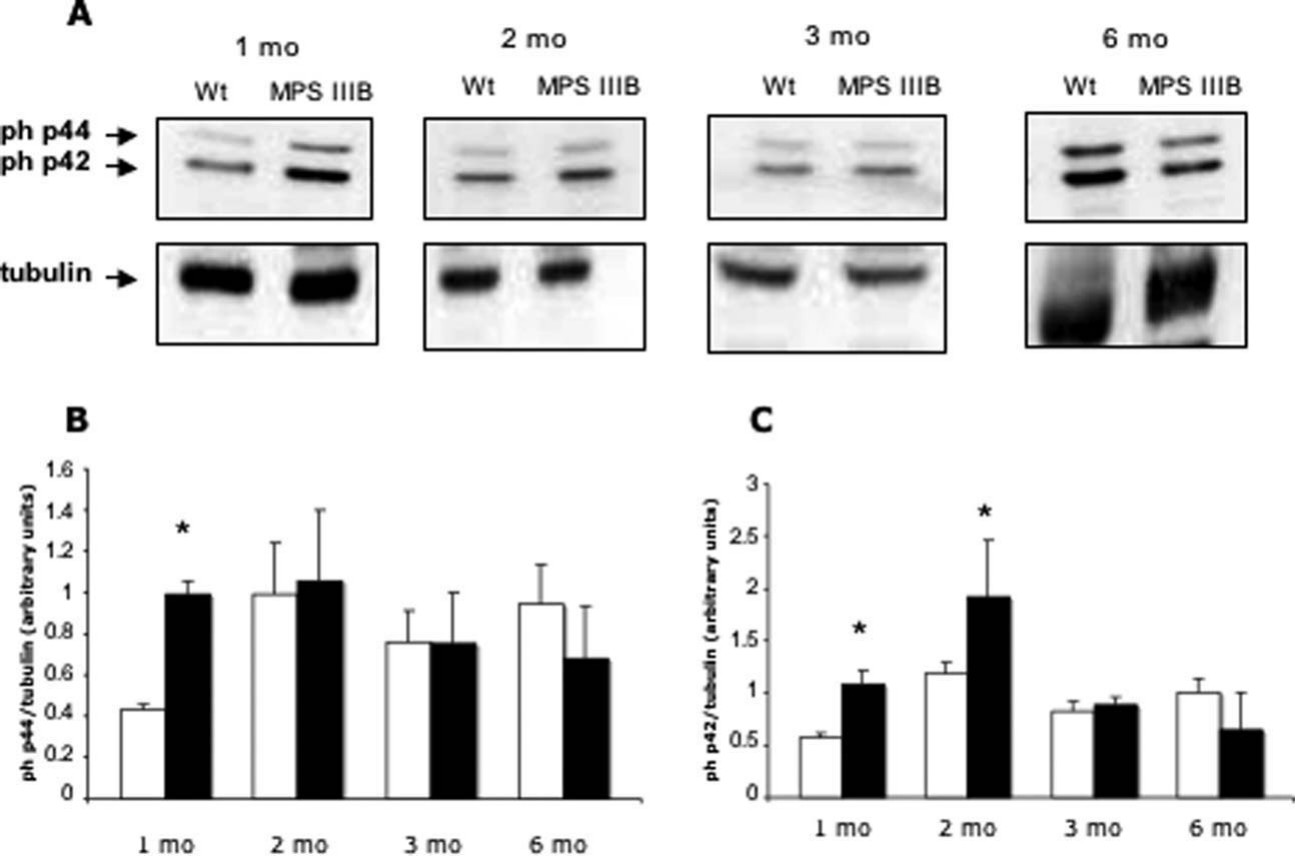
ERK is activated in the cortex of 1-month-old MPS IIIB mice. Activation of ERK was visualized using specific antibody for its phosphorylated form. **A:** Representative Western blot analysis of phospho-ERK1/2 (p44/p42) on homogenates of the cortex of Wt and MPS IIIB mice. Western blot analysis of tubulin, performed on the same gel, is shown for comparison. ERK phosphorylation was quantitated after scanning as ratio between p-ERK1 and tubulin (**B**) and between p-ERK2 and tubulin (**C**). Each band density was calculated separately. The histogram shows the mean ± SD of five independent experiments. The levels of phospho-p44 and phospho-p42 in 1–2-month-old MPS IIIB mice were significantly higher than in Wt (*1-month-old *P* < 0.005; *2-month-old *P* < 0.1, Student's *t*-test). Open bars, Wt mice, n = 5; solid bars, affected mice, n = 5.

We evaluated the activation status of ERK1/2 in the cortex of MPS IIIB and Wt mice by Western blot on the homogenates of cortex derived from mice at different ages. We used a primary monoclonal antibody raised against phospho-(Thr202/Tyr204)-ERK, selective for the two isoforms of the enzyme, corresponding to phospho-ERK1 and phospho-ERK2, with apparent molecular weights of 44 kDa and 42 kDa, respectively. We found that both p44 and p42 were significantly activated in 1–2-month-old MPS IIIB mice, as shown in Figure 1. The quantitative analysis performed comparing the density of the corresponding bands with that of tubulin is shown in Figure 1B,C, respectively. The levels of both p44 and p42 in MPS IIIB mice (Fig. 1, solid bars) were twofold higher than in Wt littermates (Fig. 1, open bars), and the increase was statistically significant. Selective up-regulation of ERK1/2 was lost with older mice (3 and 6 months).

**Fig. 2:**
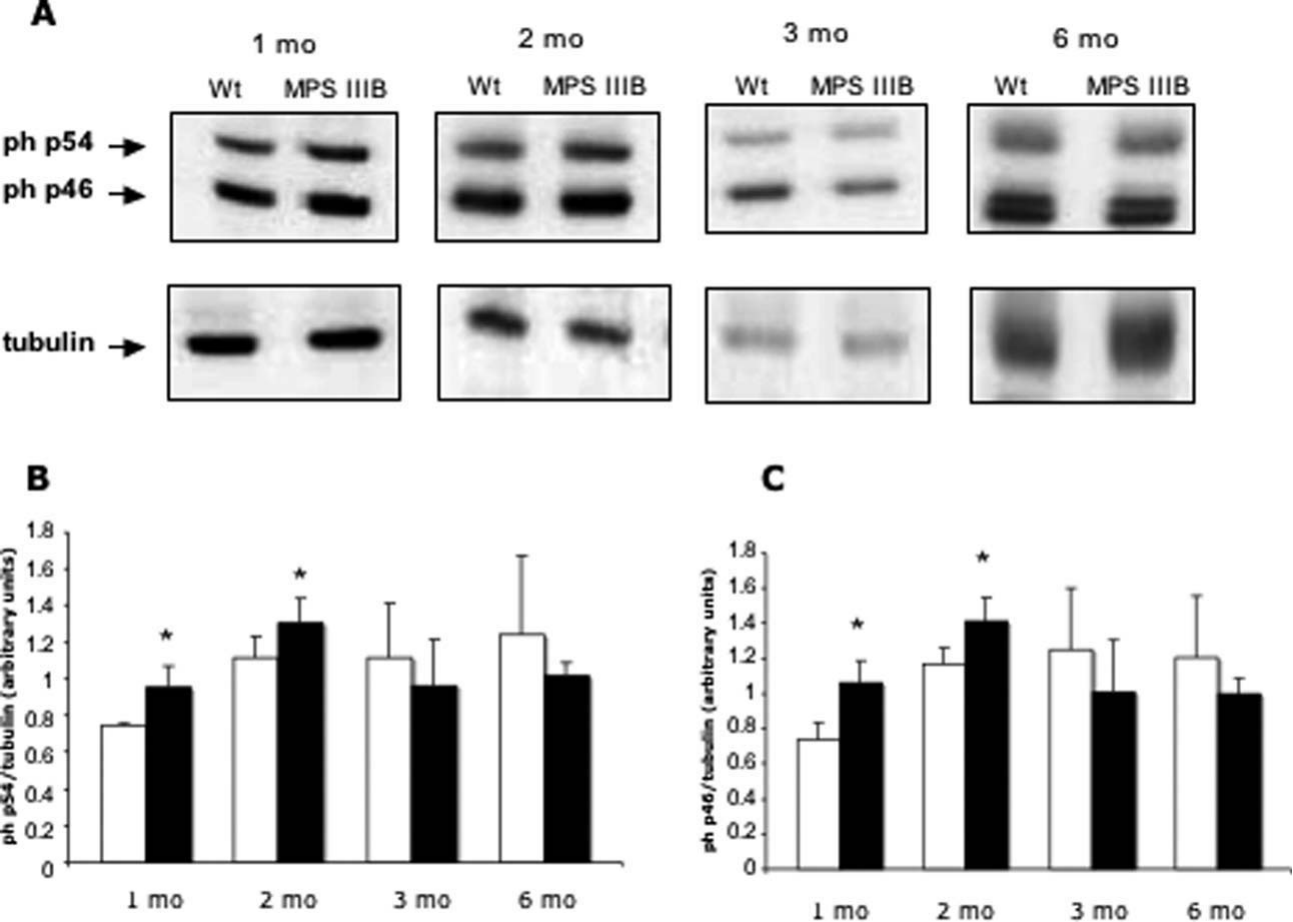
Activation of SAPK/JNK in the cortex of 2-month-old MPS IIIB mice. Activation of SAPK/JNK was visualized using specific antibody for its phosphorylated form. **A:** Representative Western blot analysis of phospho-SAPK/JNK (p54/p46) on homogenates of the cortex of Wt and MPS IIIB mice; Western blot analysis of tubulin, performed on the same gel, is shown for comparison. B,C: Quantitative analysis of activated SAPK/JNK performed comparing the immunopositive band of phospho-p54 (**B**) or phospho-p46 (**C**) with that of tubulin run on the same gel; each band density was calculated separately. The histogram values shows the mean ± SD of five independent experiments. The levels of phospho-p46 in 1–2-month-old MPS IIIB mice were significantly higher than in Wt (*1-month-old *P* < 0.1; *2-month-old *P* < 0.05, Student's *t*-test); Open bars, Wt mice, n = 5; solid bars, affected mice, n = 5.

The activation status of SAPK/JNK was evaluated by Western blot using a polyclonal antibody raised against phospho-(Thr183/Tyr185)-SAPK-JNK, showing only two major bands at apparent molecular weights of 54 kDa and 46 kDa. Figure 2A indicates that SAPK-JNK is activated in the first 2 months from birth of MPS IIIB mice compared with Wt controls. The quantitative analyses performed comparing the density of the corresponding bands with that of tubulin are illustrated in Figure 2B,C, respectively. A significant increase was evident only at 1–2 months of age. As for pERK1/2, the selective up-regulation of JNK decreased with age.

### Down-Regulation of p38MAPK in the Cortex of MPS IIIB Mice

**Fig. 3:**
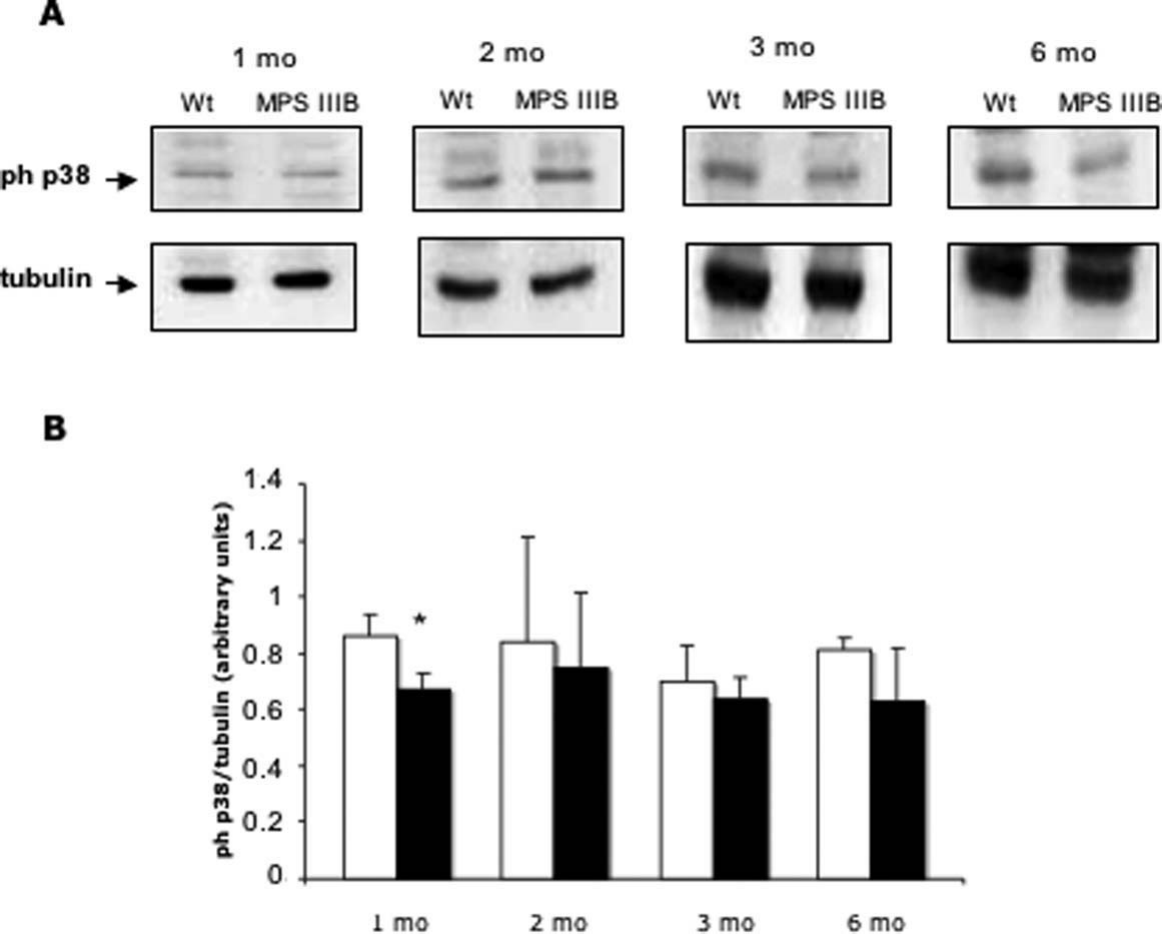
Down-regulation of p38MAPK in the cortex of 1-month-old MPS IIIB mice. Activation of p38MAPK was visualized using specific antibody for its phosphorylated form. **A:** Representative Western blot analysis of phospho-p38MAPK on homogenates of the cortex of Wt and MPS IIIB mice. Western blot analysis of tubulin, performed on the same gel, is shown for comparison. **B:** Quantitative analysis of activated p38MAPK performed comparing the immunopositive band of phospho-p38MAPK with that of tubulin run on the same gel. The histogram shows the mean ± SD of five independent experiments. The levels of phospho-p38MAPK in 1-month-old MPS IIIB mice were significantly lower than in Wt (* *P* < 0.05, Student's *t*-test). Open bars, Wt mice, n = 5; solid bars, affected mice, n = 5.

To evaluate the activation status of p38MAPK, a Western blot was performed using an antiphospho-p38MAPK antibody raised against phospho-(Thr180/Tyr182)-p38MAPK, showing a single major band at an apparent molecular weight of 38 kDa. Figure 3A shows a representative Western blot demonstrating that p38 is present in the cortex of MPS IIIB mice but is less than that found in the cortex of Wt mice. A quantitative analysis of the data found in Wt (Fig. 3, open bars) and MPS IIIB (Fig. 33, solid bars), performed by comparison with the tubulin band, is shown in Figure 3B. The data show that p38MAPK is less phosphorylated in the cortex of MPS IIIB mice at each time considered from birth, although the difference is statistically significant only at 1 month of age.

### HS Levels in Primary Cortical Neurons

Neurons were isolated from the cortex of Wt and MPS IIIB mice at embryonic day 16. The levels of HS were significantly higher (*P* < 0.005, Student's *t*-test) in cells from affected mice (8.3 ± 1.1 μg GAGs/mg protein) with respect to those found in Wt controls (2.8 ± 0.65 μg GAGs/mg protein).

### Phosphorylation Status of MAPKs in Cultured Neurons From MPS IIIB Mice

**Fig. 4:**
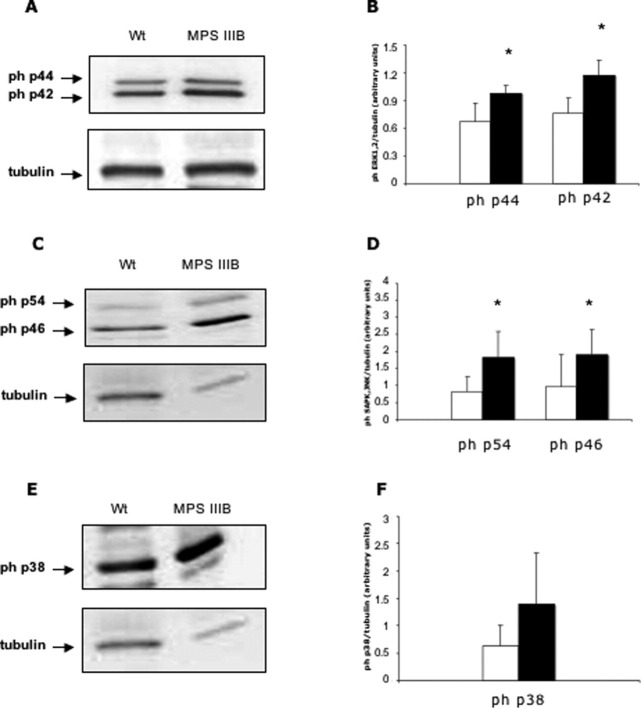
Activation of MAPKs in cultured neurons from MPS IIIB mice. Phosphorylation status of MAPKs ERK1/2, SAPK/JNK, and p38MAPK were visualized using specific antibody for their phosphorylated form on cellular extracts obtained from cortical neurons of MPS IIIB and Wt mice at 8 days in vitro. **A,C,E:** Representative Western blot analysis of phospho-ERK1/2 (A), SAPK/JNK (C), and p38MAPK (E); Western blot analysis of tubulin performed on the same gel is shown for comparison. **B,D,F:** Quantitative analysis of activated kinases performed comparing the immunopositive band of phospho-ERK1/2 (B), SAPK/JNK (D), and p38MAPK (F) with that of tubulin run on the same gel; each band density was calculated separately. The histogram shows the mean ± SD of five independent experiments.The levels of phospho-p44 and phospho-p42 in cultured neurons from MPS IIIB mice were significantly higher than in Wt (p44 * *P* < 0.1 and p42 * *P* < 0.05, Student's *t*-test); the levels of phospho-p54 and phospho-p46 (* *P* < 0.1, Student's *t*-test) were also higher. No significant differences were observed for p38 kinase. Open bars, Wt mice, n = 5; solid bars, affected mice, n = 5.

To evaluate whether the alterations in the MAPK pathway identified in the cortex of MPS IIIB mice are present already at an early stage of cortex development, we analyzed the phosphorylation status of MAPKs in neurons cultured from MPS IIIB and Wt mice. The activation of MAPKs in cultured neurons was analyzed by Western blotting on protein extract obtained from MPS IIIB and Wt cortical neurons at 8 DIV. Both ERK and SAPK/JNK showed an increase in the phosphorylated bands in MPS IIIB neurons ( Fig. 4A,C). A quantitative analysis performed comparing the immunopositive bands of each kinases with that of tubulin reveals that the differences between MPS IIIB (Fig. 4, solid bars) and Wt (Fig. 4, open bars) are statistically significant ( Fig. 4B,D). Increased phosphorylation of the p38MAPK band seems to be present in MPS IIIB cultured neurons ( Fig. 4E), but no statistical significance was shown by quantitative analysis ( Fig. 44F).

## DISCUSSION

In the present work, we studied the activation of the three main stress kinases (MAPK) in an MPS IIIB mouse model and showed that a selective activation of MAPK is involved in the disease pathogenesis. Our data show an increased activation of ERK and SAPK/JNK in the first 2 months after birth in the cortex of MPS IIIB animals. Although the increased ERK and SAPK/JNK expression was detected in the cortex, at present we cannot exclude that it could also be present outside the cortical area. Our results are in agreement with the findings of early activation of ERK in some neurodegenerative diseases: phosphorylated ERK immunoreactivity has been found in neurons in Pick's disease and progressive supranuclear palsy and corticobasal degeneration (Ferrer et al.,^2001^), in hippocampal neurons with neurofibrillar degeneration in Alzheimer's disease (AD; Perry et al.,^1999^), and in astrocytes from early AD patients (Webster et al.,^2006^). In all these cases, ERK activation was not maintained with disease progression.

The alterations in the CNS in MPS IIIB shared some features with other neurodegenerative diseases, especially Alzheimer's disease (AD), such as the accumulation of abnormal products, activation of microglia, and alteration of the lysosomal and autophagic degradation pathway. In this regard, it is interesting to note that Ohmi and coworkers (2009) recently described for the first time the presence of a hyperphosphorylated form of microtubule-associated protein tau in the brain of MPS IIIB mice, a characteristic of many storage diseases associated with dementia. An accumulation of HS is the primary consequence of the enzyme defect in MPS IIIB and triggers a cascade of pathological events that progressively leads to CNS pathology. Ausseil and coworkers (2008) detected GAG accumulation in the brain of MPS IIIB mice already at the age of 10 days, but we observed the HS accumulation in neuron cultures from the brain of E16 MPS IIIB mice. It has been demonstrated that heparin is able to promote tau aggregation in vitro, so it is likely that this function is carried out by HS in vivo (Pandel and Li,^1999^). This observation could suggest that HS accumulation in the early stage of MPS IIIB disease could promote tau aggregation and consequent hyperphosphorylation through the activation of MAPK, as demonstrated in other models of neurodegeneration (Perez et al.,^2008^). Alternatively, early activation of ERK and SAPK/JNK may represent a first response to the onset of MPS IIIB pathology and thus may be linked to neuroprotective pathways. In fact, as discussed above, different kinetics of ERK activation can account for its dual effects, i.e., survival or death of neuronal cells (Colucci-D'Amato et al.,^2003^). Interestingly, the rises in pERK and pSAPK/JNK immunoreactivity were transient and more characteristic of the early stage of disease and, therefore, may be a reaction to ongoing neuronal damage. Thus, the increase in ERK and SAPK/JNK phosphorylation may be a reaction to microglia activation caused by HS, a feature that Ausseil and coworkers (2008) identified in the early stage of MPS IIIB brain pathology.

Our results from the cortex of MPS IIIB mice were confirmed by the study of embryonic cultured cortical neurons, in which the patterns of ERK and SAPK/JNK phosphorylation reflect those observed in the brain of postnatal mice and permit us to speculate that this activation is present already during fetal life. Moreover, our data obtained from pure neuronal cultures, devoid of astrocytes and microglia, allow us reasonably to assume that this phenomenon is not secondary to the activation of microglia, and this is in agreement with the assumption of Ausseil and coworkers (2008) that, in the early stage of MPS IIIB disease, the pathology of the CNS is cell autonomous rather than environmental.

We found that p38MAPK was down-regulated in the cortex of MPS IIIB mice compared with Wt littermates. Increased activation of p38MAPK has been found in brain homogenates in all the tauopathies (Ferrer et al.,^2005^), and often phosphorylated p38MAPK immunoreactivity has been found in colocalization with activated SAPK/JNK (Ferrer et al.,^2001^; Giovannini et al.,^2008^). It is widely accepted that the activation of p38MAPK is related to the production of proinflammatory cytokines but also to an aggravation of the inflammatory processes, a feature shared by most chronic neurodegenerative diseases. The inhibition of p38MAPK phosphorylation could support the hypothesis that early activation of ERK and SAPK/JNK represents a first response to the onset of the pathology.

In conclusion, these experiments show that a dysregulation of MAPK is present in the murine model of MPS IIIB. In particular, the early stages of the disease are characterized by simultaneous activation of ERK and SAPK/JNK pathways and inhibition of p38MAPK phosphorylation. These results together with our findings from pure neuronal cultures allow us to suggest not only that the alteration of the MAPK pathways is involved in the pathophysiological changes that occur early on in the brain of MPS IIIB mice but also that these events are cell autonomous and not dependent on environmental alterations, at least in the early stages of brain disease.

We propose that, in the early pathological mechanisms involved in MPS IIIB disease, a dysregulation of stress kinases could be included in the pathogenetic cascade following enzymatic deficit. It could well be, in fact, that an increased ROS production (Villani et al.,^2007^, ^2009^) might lead to the activation of ERK1/2 and SAPK/JNK and to the inhibition of p38MAPK (this work). However, whether this leads to cell death, as suggested for other lysosomal diseases (for review see Walkley,^2009^), remains to be demonstrated for MPS IIIB. In this regard, previous data on apoptotic or nonapoptotic mechanisms in brain disease of MPS IIIB are controversial (Li et al.,^2002^; Villani et al.,^2007^, ^2009^). Finally, although further studies are required to define the exact sequence of pathological events in MPS IIIB brain disease, our work, to our knowledge, is the first study reporting that cell signaling transduction alterations are involved in the MPS IIIB neuronal pathology.

## References

[b1] Ausseil J, Desmaris N, Bigou S, Attali R, Corbineau S, Vitry S, Parent M, Cheillan D, Fuller M, Maire I, Vanier MT, Heard JM (2008). Early neurodegeneration progress independently of microglial activation by heparan sulfate in the brain of mucopolysaccharidosis IIIB mice. PloS One.

[b2] Bjornsson S (1993). Simultaneus preparation and quantitation of proteoglycans by precipitation with Alcian blue. Anal Biochem.

[b3] Bradford MM (1976). A rapid and sensitive method for the quantitation of microgram quantities of protein utilizing the principle of protein dye binding. Anal Biochem.

[b4] Colucci-D'Amato L, Perrone-Capano C, di Porzio U (2003). Chronic activation of ERK and neurodegenerative diseases. Bioessays.

[b5] Cressant A, Desmaris N, Verot L, Brejot T, Froissart R, Vanier MT, Maire I, Heard JM (2004). Improved behavior and neuropathology in the mouse model of Sanfilippo type IIIB disease after adeno-associated virus-mediated gene transfer in the striatum. J Neurosci.

[b6] Dhillon AS, Hagan S, Rath O, Kolch W (2007). MAP kinase signalling pathways in cancer. Oncogene.

[b7] Di Domenico C, Villani GR, Di Napoli D, Nusco E, Calì G, Nitsch L, Di Natale P (2009). Intracranial gene delivery of LV-NAGLU vector corrects neuropathology in murine MPS IIIB. Am J Med Genet.

[b8] Di Natale P, Di Domenico C, Gargiulo N, Castaldo S, Gonzales y Reyero E, Mithbaokar P, De Felice M, Follenzi A, Naldini L, Villani GR (2005). Treatment of the mouse model of mucopolysaccharidosis type IIIB with lentiviral-NAGLU vector. Biochem J.

[b9] DiRosario J, Divers E, Wang C, Etter J, Charrier A, Jukkola P, Auer H, Best V, Newsom DL, McCarty DM, Fu H (2009). Innate and adaptive immune activation in the brain of MPS IIIB mouse model. J Neurosci Res.

[b10] Ferrer I, Blanco R, Carmona M, Ribera R, Goutan E, Puig B, Rey MJ, Cardozo A, Vinals F, Ribalta T (2001). Phosphorylated map kinase (ERK1, ERK2) expression is associated with early tau deposition in neurones and glial cells, but not with increased nuclear DNA vulnerability and cell death, in Alzheimer disease, Pick's disease, progressive supranuclear palsy and corticobasal degeneration. Brain Pathol.

[b11] Ferrer I, Gomez-Isla T, Puig B, Freixes M, Ribé E, Dalfó E, Avila J (2005). Current advances on different kinases involved in tau phosphorylation, and implications in Alzheimer's disease and tauopathies. Curr Alzheimer Res.

[b12] Giovannini MG, Cerbai F, Bellucci A, Melani C, Grossi C, Bartolozzi C, Nosi D, Casamenti F (2008). Differential activation of mitogen-activated protein kinase signalling pathways in the hyppocampus of CRND8 transgenic mouse, a model of Alzheimer's disease. Neuroscience.

[b13] Haddad F, Adams GR (2004). Inhibition of MAP/ERK kinase prevents IGF-I-induced hypertrophy in rat muscles. J Appl Physiol.

[b14] Kim EK, Choi EJ (2010). Pathological roles of MAPK signaling pathways in human diseases. Biochim Biophys Acta.

[b15] Li HH, Zao HZ, Neufeld EF, Cai Y, Gomez-Pinilla F (2002). Attenuated plasticity in neurons and astrocytes in the mouse model of Sanfilippo syndrome type B. J Neurosci Res.

[b16] McCubrey JA, Lahair MM, Franklin RA (2006). Reactive oxygen species-induced activation of the MAP kinase signaling pathways. Antiox Redox Signal.

[b17] Neufeld EF, Muenzer J, Scriver CR, Beaudet AL, Sly WS, Valle D (2001). The mucopolysaccaridoses. The metabolic and molecular bases of inherited disease.

[b18] Ohmi K, Greenberg DS, Rajavel KS, Ryazantsev S, Li HH, Neufeld EF (2003). Activated microglia in cortex of mouse models of mucopolysaccaridosis I and III B. Proc Natl Acad Sci U S A.

[b19] Ohmi K, Kudo LC, Ryazantsev S, Zhao HZ, Karsten SL, Neufeld EF (2009). Sanfilippo syndrome type B, a lysosomal storage disease, is also a tauopathy. Proc Natl Acad Sci U S A.

[b20] Paudel HK, Li W (1999). Heparin-induced conformational change in microtuble-associated protein Tau as detected by chemical cross-linking and phosphopeptide mapping. J Biol Chem.

[b21] Perez M, Moràn MA, Ferrer I, Avila J, Gòmez-Ramos P (2008). Phosphorylated tau in neuritic plaques of APP(sw)/tau(vlw) transgenic mice and Alzheimer disease. Acta Neuropathol.

[b22] Perry G, Roder H, Nunomura A, Takeda A, Friedlich AL, Zhu X, Raina AK, Holbrook N, Siedlak SL, Harris PL, Smith MA (1999). Activation of neuronal extracellular receptor kinase (ERK) in Alzheimer disease links oxidative stress to abnormal phosphorylation. Neuroreport.

[b23] Ryazantsev S, Yu WH, Zao HZ, Neufeld EF, Ohmi K (2007). Lysosomal accumulation of SMACS (subunit c of mitochondrial ATP synthase) in neurons of the mouse model of mucopolysaccharidosis IIIB. Mol Genet Metab.

[b24] Scorziello A, Pellegrini C, Forte L, Tortiglione A, Gioielli A, Iossa S, Amoroso S, Tufano R, Di Renzo G, Annunziato L (2001). Differential vulnerability of cortical and cerebellar neurons in primary culture to oxygen glucose deprivation followed by reoxygenation. J Neurosci Res.

[b25] Seger R, Krebs EG (1995). The MAPK signaling cascade. FASEB J.

[b26] Subramaniam S, Zirrgiebel U, von Bohlen Und Halbach O, Strelau J, Lalibertè C, Kaplan DR, Unsicker K (2004). ERK activation promotes neuronal degeneration predominantly through plasma membrane damage and independently of caspase-3. J Cell Biol.

[b27] Villani GR, Gargiulo N, Faraonio R, Castaldo S, Gonzales y Reyero E, Di Natale P (2007). Cytokines, neurotrophins, and oxidative stress in brain disease from mucopolysaccharidosis IIIB. J Neurosci Res.

[b28] Villani GR, Di Domenico C, Musella A, Cecere F, Di Napoli D, Di Natale P (2009). Mucopolysaccharidosis IIIB: oxidative damage and cytotoxic cell involvement in the neuronal pathogenesis. Brain Res.

[b29] Vitry S, Ausseil J, Hocquemiller M, Bigou S, Dos Santos Coura R, Heard JM (2009). Enhanced degradation of synaptophysin by the proteasome in mucopolysaccharidosis type IIIB. Mol Cell Neurosci.

[b30] Walkley SU (2009). Pathogenetic cascades in lysosomal disease—Why so complex?. J Inherit Metab Dis.

[b31] Webster B, Hansen L, Adame A, Crews L, Torrance M, Thal L, Masliah E (2006). Astroglial activation of extracellular-regulated kinase in early stages of Alzheimer disease. J Neuropathol Exp Neurol.

[b32] Willaime-Morawek S, Brami-Cherrier K, Mariani J, Caboche J, Brugg B (2003). C-Jun N-terminal kinase/c-Jun and p38 pathways cooperate in ceramide-induced neuronal apoptosis. Neuroscience.

[b33] Zheng Y, Ryazantsev S, Ohmi K, Zhao HZ, Rozengurt N, Kohn DB, Neufeld EF (2004). Retrovirally transduced bone marrow has a therapeutic effect on brain in the mouse model of mucopolysaccharidosis IIIB. Mol Genet Metab.

[b34] Zhu X, Raina AK, Rottkamp CA, Aliev G, Perry G, Boux H, Smith MA (2001). Activation and redistribution of c-Jun N-terminal kinase/stress activated protein kinase in degenerating neurons in Alzheimer's disease. J Neurochem.

